# Effect of selenium and compost on physiological, biochemical, and productivity of chili under chromium stress

**DOI:** 10.1038/s41598-025-95012-y

**Published:** 2025-03-24

**Authors:** Fahad Ahmad, Sabiha Javied, Kamran Ashraf, Aamir Amanat Ali Khan, Zeeshan Ahmed, Khawar Sultan, Ijaz Ali, Qamar uz Zaman, Ghulam Murtaza, Abd El-Zaher M. A. Mustafa, Mohamed S. Elshikh, Rashid Iqbal, Nazim S. Gruda

**Affiliations:** 1 Department of Environmental Sciences, The University of Lahore, Lahore, 54590 Pakistan; 2 State Key Laboratory of Bioreactor Engineering, East China University of Science and Technology, Shanghai, 200237 People’s Republic of China; 3 Xinjiang Institute of Ecology and Geography, Chinese Academy of Sciences, Ürümqi, 830011 People’s Republic of China; 4 Cele National Station of Observation and Research for Desert-Grassland Ecosystems, Chinese Academy of Sciences, Ürümqi, 848300 People’s Republic of China; 5 College of Life Science, Shenyang Normal University, Shenyang, 110034 People’s Republic of China; 6 Centre for Applied Mathematics and Bioinformatics, Gulf University for Science and Technology, 32093 Hawally, Kuwait; 7 School of Agriculture, Yunnan University, Kunming, 650504 Yunnan People’s Republic of China; 8 Department of Botany and Microbiology, College of Science, King Saud University, P.O. 2455, 11451 Riyadh, Saudi Arabia; 9 Department of Agronomy, Faculty of Agriculture and Environment, The Islamia University of Bahawalpur, Bahawalpur, 63100 Pakistan; 10 Department of Life Sciences, Western Caspian University, Baku, Azerbaijan; 11 Division of Horticultural Sciences, Institute of Plant Sciences and Resource Conservation, University of Bonn, 53115 Bonn, Germany

**Keywords:** Biochemical, Antioxidant enzymes, Lipid per oxidate, Biomass, Combination of application of compost with selenium, Plant sciences, Environmental sciences

## Abstract

In the era of industrialization, chromium (Cr) as a metal poses a substantial threat to the ecosystem. Selenium (Se) is essential for minimizing heavy metal stress in crops and effectively reducing their accumulation in edible plant parts. This research work aimed to evaluate the synergistic effect of compost and Se for alleviation of Cr stress in chili plants. A greenhouse trail was conducted to investigate the individual and combined effects of foliage applied selenium (Na_2_SeO_4_ = 3 µM) and soil applied compost (250 mg kg^−1^, w/w) on the growth, physio-biochemical, antioxidant and of chili grown under varying levels of induced Cr stress (0, 100, and 200 mg kg^−1^ using K_2_Cr_2_O_7_). Findings revelaed that a significant linear reduction was observed in growth, biomass, and physiological parameters of chili plant with an increasing level of Cr concentrations. Maximum decrease in relative water content (20.04 and 27.21%) and total chlorophyll concentrations (11.73 and 20.57%) and increased in electrolyte leakage (59.14 and 130.52%) was observed at 100 and 200 mg kg^−1^ of Cr levels in soil in comparison with the control, respectively. Combined application of compost and selenium showed significant increase regardless of Cr concentrations in the soil. Synergistic approach of compost and selenium showed improved growth in comparison with the sole application in limiting the movement and uptake of Cr in the roots and fruits of chili plants. Moreover, improved physiological and antioxidant potential of chili plants helped to cope with higher levels of Cr stress by limiting the lipid peroxidation and membrane damange. The combined use of compost and selenium induces the physio-biochemical defense responses against of the varying levels of Cr stress in chili plants. This promising approach highlights the significant potential for growing chili crop in Cr-contaminated soils to achieve better quality and higher yields.

## Introduction

The cultivation of chili holds significant agricultural and economic value in Pakistan, serving as a fundamental staple crop for domestic consumption as well as a key commodity for export. In Pakistan, it is widely recognized as “red chili” and holds significant importance in local culinary practices^1^. The cultivation of chili has a significant role in supporting the livelihoods of number of farmers residing in rural areas. The cultivation of this particular crop is frequently undertaken for commercial purposes, due to its supreme demand in both local and international markets^2^. Similar to several agricultural commodities, chili farming encounters various obstacles including pest infestations, utilization of wastewater for irrigation, soil contamination, and eratic changes in climatic patterns^3,4^. The higher levels of Cr can also interfere with the plant’s metabolic activities, disrupting photosynthesis and chlorophyll production. This leads to chlorosis, yellowing leaves, and a decline in the plant's ability to capture and convert sunlight into energy^5^. Consequently, there is a decrease in chlorophyll content, which further exacerbates the plant's reduced growth and overall productivity. The Cr toxicity hinders growth and accumulates Cr metal in edible parts, posing a threat to food safety for the consumers^6^.

Chromium (Cr) contamination in soil is a persistent environmental concern with far-reaching consequences. It is a naturally occurring element found in various forms, including trivalent (Cr (III)) and hexavalent (Cr (VI)), with the latter being more toxic and mobile in soil^7^. Sources of Cr contamination include industrial activities, such as metal plating, leather tanning, manufacturing, and the disposal of hazardous waste. It can persist for extended periods, posing threats to the environment and human health^8^. In soils the Cr contamination significantly affect plant growth, as it impacts various physiological and biochemical processes essential for the health of plant. From the literature it is evident that Cr is toxic metal that significantly restrict the growth and productivity of plants, Cr toxicity has been reported in plant nutrient solution (0.5–5.0 mg L^−1^) and in soil (5–100 mg g^−1^)^9–11^. Less than 2 g of hexavalent Cr intake can deteriorate the kidney and liver within the exposure of four days, whereas 2–5 g of Cr in hexavalent form is toxic for humans^12^. Moreover, the accumulation of chromium in edible parts of filed crops poses a significant health risk to consumers, as it can lead to the ingestion of toxic levels of chromium, potentially causing serious health issues^13^. The remediation of Cr-contaminated soil is a complex challenge due to the persistence and mobility of chromium in the environment, which requires advanced and often costly techniques such as phytoextraction, bioremediation, and chemical immobilization to mitigate Cr contamination^14^.

Compost and selenium (Se) have emerged as promising strategies to alleviate Cr contamination in field crops, by enhancing soil quality and providing essential nutrients that helps to mitigate chromium stress^15^. Compost is rich in organic matter and essential nutrients, and enhances soil structure and microbial activity^16,17^. Application of compost in Cr-contaminated soil acts as a natural chelating agent, binding with Cr ions and reducing their bioavailability^17^. Additionally, selenium often considered a beneficial trace element, can reduce the toxicity of Cr by modifying its chemical forms. Se-enriched soils stimulate the conversion of toxic Cr (VI) into less harmful Cr (III) forms, which are less mobile and less readily absorbed by plants^18^. Including glutathione peroxidase Se is essential component of antioxidant enzymes. These enzymes are important in alleviating the potential oxidative stress associated with free radicals that are generally produced due to accumulation of Cr. Se also forms complexes with Cr, resulting in lowering the bioavailability of Cr and consequently also limiting its absorption and toxic effects. Combined application of compost with Se treatments synergistically enhances their effectiveness in mitigating Cr contamination, as compost improves soil conditions and nutrient availability, while Se aids in the transformation of Cr (VI) into a less toxic state^19^.

Addressing Cr contamination in soil is crucial for protecting ecosystems, ensuring the safety and sustainability of agriculture, and protecting human health from the potential adverse effects of Cr exposure^20^. Additionally, compost can raise nutrient absorption and increase root growth synergistically supporting the application of Se in reinforcing antioxidant defense system and detoxification pathways in crops^21,22^. However, there are a few studies that have reported the synergistic effect which refers to the interaction of compost and Se to produce a combined effect more significant than the sum of their separate effects for the mitigation of Cr contamination in terms of morpho-physio-biochemical characteristics in chili plants. Our hypothesis suggests that the combined application of Se and compost can regulate the higher levels of Cr by enhancing plant growth and development by limiting the uptake of Cr metal in the plant continuum. From this perspective, the aims of this research were to explore the impact of varying levels of Cr stress on the growth, biomass and Cr bioaccumulation in chili plants, as well as to assess the individual and combined effect of Se and compost on the morpho-physio-biochemical parameters of chili plants, both in the presence and absence of chromium. This research provides valuable insights into sustainable and eco-friendly approaches for managing Cr contamination in chili cultivation, which not only protect crop yields but also ensure food safety and environmental sustainability.

## Materials and methods

### Experimental design,treatments and growth conditions

This green-house study was conducted at the Department of Environmental Sciences, The University of Lahore, Pakistan. The chili pepper seeds were purchased from the Regional Agricultural Research Institute (RARI) Bahawalpur, Pakistan. Experiment was laid out under completely randomized design (CRD) with factorial arrangement replicated thrice. The study was comprised of experimental units (2 × 4 × 3 = 24). Experimental treatments were comprised of two factors: factor A; chromium stress viz., control, 100 and 200 mg kg^−1^ (w/w) using K_2_Cr_2_O_7_ as Cr source^23^ and factor B; selenium and compost treatments viz., control; selenium (3 µM, using Na_2_SeO_4_ as Se source by Sigma-Aldrich (USA) with 98% purity)^24^; compost (250 mg kg^−1^ w/w)^25^ and a mixture of selenium and compost. Throughout the experimental period from last week of February to first week of June, average average day/night temperature was 29 ± 5/17 ± 2 °C and, average relative humidity 60 ± 3%.

### Crop management

Soil samples in this experimental work were collected from the agricultural area at a depth of about 15 cm. Soil samples were air dried under indoor conditions until soil lumps could be crumbled into smaller sizes to pass through two set sieves, first from 4 mm and then from 2 mm size sieves, for further use in the pot experiment. Soil textural analysis showed that 30% sand, 35% silt, and 35% clay contents in the soil. The pH of soil 7.89, electrical conductivity 0.18 mS cm^−1^, organic matter content 0.79%, available phosphorus (29 mg kg^−1^) and available potassium (28 mg kg^−1^).For induction of heavy metal content related stress treatments, potassium dichromate (K_2_Cr_2_O_7_) was mixed with the soil medium at three different concentrations (0, 100, and 200 mg kg^−1^) and incubated for 3 weeks. After the incubation period the soil mixed with the Cr treatments filled in the plastic pots having dimension (diameter of 20 cm and depth of 18 cm). After the addition of compost as per treatments manual hoeing was done for three days for the homogenization of the compost with the soil. The chili pepper seeds were germinated in peat under the temperature of 25 °C for ten days. After that, three chili seedlings were picked and transplanted into pots. Hoagland solution (50%) was applied at a rate of 1000 mL per week per pot for the nutritional reqiurements of the plants. Throughout the experiment, weeding and irrigation was carried out regularly depending upon the requirements of the plants in pots. The application of Se was done with a 500 mL solution application to each pot by a handheld sprayer with a rate of two sprays per ten-day interval^26^. Commercial detergent (1.00 mg L^−1^) as surfactant was applied to each pot with desired levels of Se solution for the purpose of adhesion of the sprayed substance to the plant following the standard procedures^27^. In this experiment, a plant set with no Cr, Se, and compost treatments was also included which served as a control for comparison and quality control. Upon maturation of plants, harvesting was done and data of biochemical, morphological and physiological attributes was carefully recorded.

### Methods for attribute measurement

#### Growth, phonological and biomass attributes

Three plants were selected randomly, and the roots and shoots of each plant were segregated to measure the growth and biomass parameters. The plants were cleaned with distilled water and then air dried to remove soil. With the help of analytical balance the fresh weight of the root, shoots and plant was measured. For the dry weight of the roots and shoots, they were dried for 48 h at 70 °C in oven till constant weight. Plant height, root, shoot, leaf length, and width were computed using a measuring scale. Leaf area was calculated by multiplying the chili plant’s leaf length and width and a coefficient (0.70). A vernier caliper was employed to measure the diameter of the chili plant’s stems.

#### Physio-biochemical and membrane damange attributes

Leaf samples (5 g) in triplicate were crushed for 24 h in a test tube containing 85% acetone (v/v) to extract the pigment. Using a spectrophotometer (Halo DB-20/ DB-20S, UK), the absorbance at 470, 647, and 664.5 nm was measured after centrifuging at 4000 g for 10 min at 4 °C. After quantifying the value in the supernatant, Lichtenthaler^28^ method was used to calculate the concentrations of chlorophylls *a, b,* and carotenoids. The sum of chslorophylls *a* and *b* was used to calculate total chlorophyll concentrations. The infrared gas analyzer (IRGA) by the Analytical Development Company, London, United Kingdom was used to determine the transpiration rate (*E*), photosynthetic rate (*A*) and stomatal conductance (*gs*) on a clear sunny day from upper leaves at 9 AM. From each treatment a leaf of constant size was selected for the measurement of relative water content (RWC) by using the Eq. 1 given below:

1 $$ RWC = \frac{{\left( {FW - DW} \right)}}{{\left( {TW - DW} \right)}} \times 100. $$

where TW = turgid weight; DW = dry weight; FW = fresh weight;

For estimation of RWC collected leaf samples, were wehed immediately to get the fresh weight, and then soaked them in distilled water for 3–4 h until fully turgid. After soaking, bloting was done from the leaves to remove surface moisture and weigh them again to obtain the turgid weight.

For the estimation of electrolyte leakage (EL), the following Eq. 2 was used:

2 $$ {\text{EC}} = \frac{{{\text{EC}}1}}{{{\text{EC}}2}}{ } \times 100 $$

For the estimation of EC1 and EC2 leaves samples were cut into uniform pieces. The leaves samples were placed in deionized water and incubated at room temperature for 24 h. The initial electrical conductivity (EC1) was calculated after that samples were again boiled to release all electrolytes and final conductivity (EC2) was calculated.

#### Enzymatic antioxidants activity

From each replication, the supernatant from 1 g of chili leaves that had been extracted with 50 mM phosphate buffer was centrifuged (15,000×*g* for 10 min) to ascertain the enzyme activity. For SOD activity A solution was prepared by adding (enzyme extract = 50 µL, PCR binding solution (SPB) = 500 µL), methionine = 100 µL, distilled water = 500 µL, and nitroblue tetrazolium chloride (NBT) = 50 µL). The whole mixture was left inside the cuvette for twenty minutes under a lamp, and at 560 nm readings were taken. For the estimation of POD and CAT activities, the CAT reaction mixture was prepared containing (potassium phosphate buffer = 3 mL, 0.1 mL of extract, and H_2_O_2_ in the cuvette. Using a spectrophotometer the readings were taken at 240 nm for every 20 s. For POD activity in the mixture of 1.5 mL of 5 Mm guaiacol and 1.5 mL of H_2_O_2_ about 0.1 mL of plant extract was added. At 470 nm the absorbance was taken. For one unit of CAT and POD activity, the 0.01-unit min^−1^ change in absorbance was considered. The enzyme’s specific activity was measured in enzyme units per mg of protein. For the determination of MDA, about 0.50 g leaf samples were ground in trichloroacetic acid (6%) for 15 min. The readings were noted at 532 and 600 nm^29–31^.

#### Chromium accumulation

About 0.5 g of chili fruit in triplicate were digested by a di-acid solution. The levels of Cr^+2^ in the chili roots and fruit were detected using a flame atomic absorption spectrophotometer (HITACHI Z-2000).

### Data analysis

The measured data was subjected to statistical analysis using Fisher's ANOVA (Analysis of Variance) technique. Treatments means were compared using the honestly significant differences (HSD) test in statistix software (version 8.1). The statistical analysis, which included correlation, and principal component analysis, and heat map analysis were done using RStudio software.

## Results

### Growth and phonological attributes

**Fig. 1:**
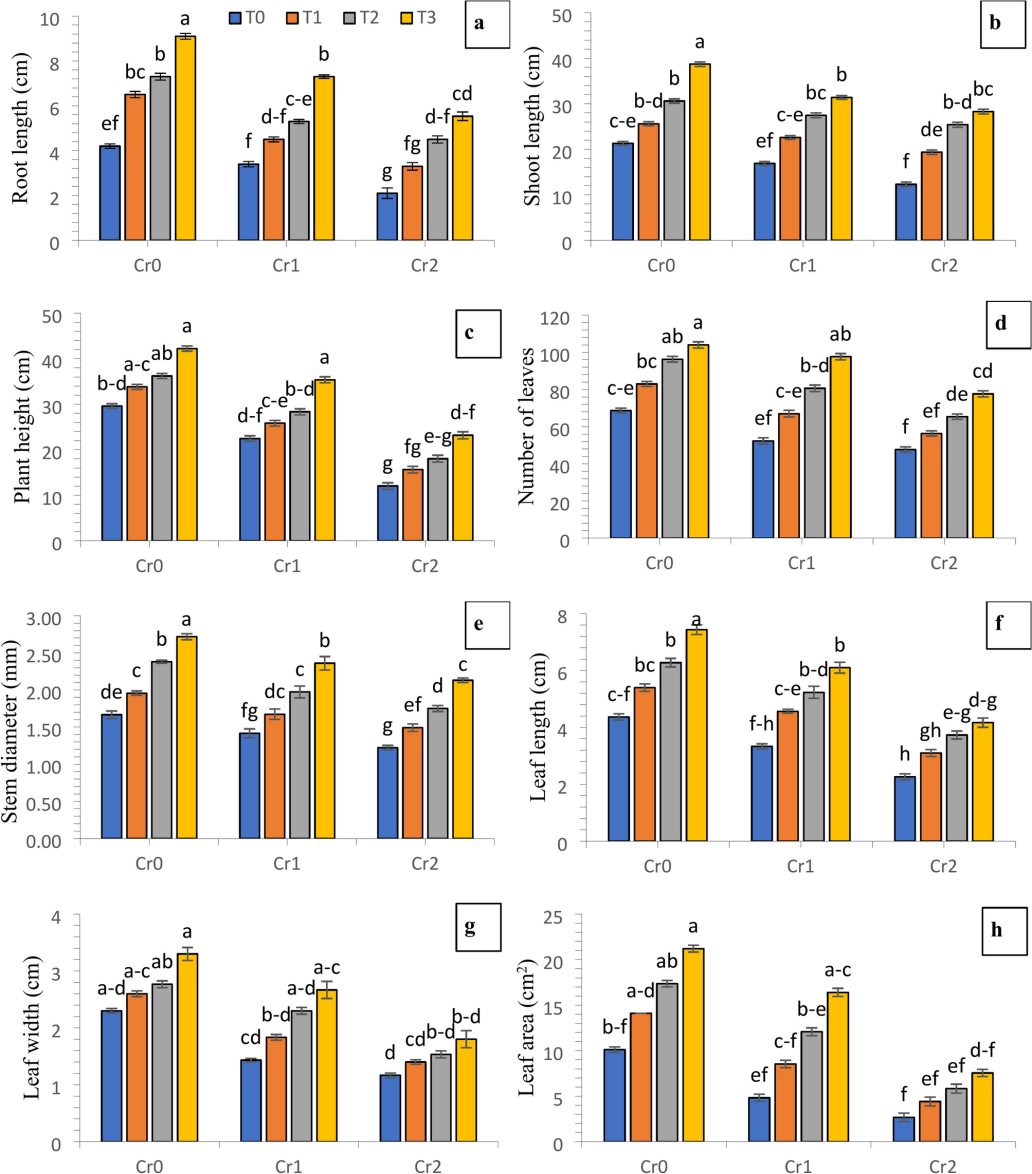
Synergistic effect of compost and Se on the growth attributes of chili plants grown under different levels of induced chromium (Cr_0_ = 0 mg kg^−1^, Cr_1_ = 100 mg kg^−1^ and Cr_2_ = 200 mg kg^−1^). Different lowercase letters represent the statistically significant difference across treatment means at *p* < 0.05. For each parameter, means with the same letter are not statistically different across treatments. T_0_ = Control; T_1_ = Se (3 µM); T_2_ = Compost (250 mg kg^−1^ of soil); T_3_ = T_1_ + T_2_; (**a**) root length; (**b**) shoot length; (**c**) plant height; (**d**) number of leaves; (**e**) stem diameter; (**f**) leaf length; (**g**) leaf width; (**h**) leaf area.

Various levels of induced Cr stress resulted in a maximum decrease in chili plant height (21.03 and 51.43%), root length (24.35 and 43.05%), shoot length (15.35 and 26.57%), number of leaves (15.44 and 29.83%), leaf length (18.07 and 43.49%), leaf width (24.92 and 46.20%), leaf area (33.35 and 67.40%) and stem diameter (14.87 and 24.35%) at 100 and 200 mg kg^−1^ of Cr levels, respectively in comparsion with control (no chromium) in chili plants. Applying compost to the soil and foliar application of Se improves the growth and phonological attributes of chili plants in both the control and Cr amended treatments. The highest level of growth and phonological attributes were observed when compost and Se were applied together in both the control and Cr polluted treatments (Fig. 1).

### Fresh and dry biomass attributes

**Fig. 2:**
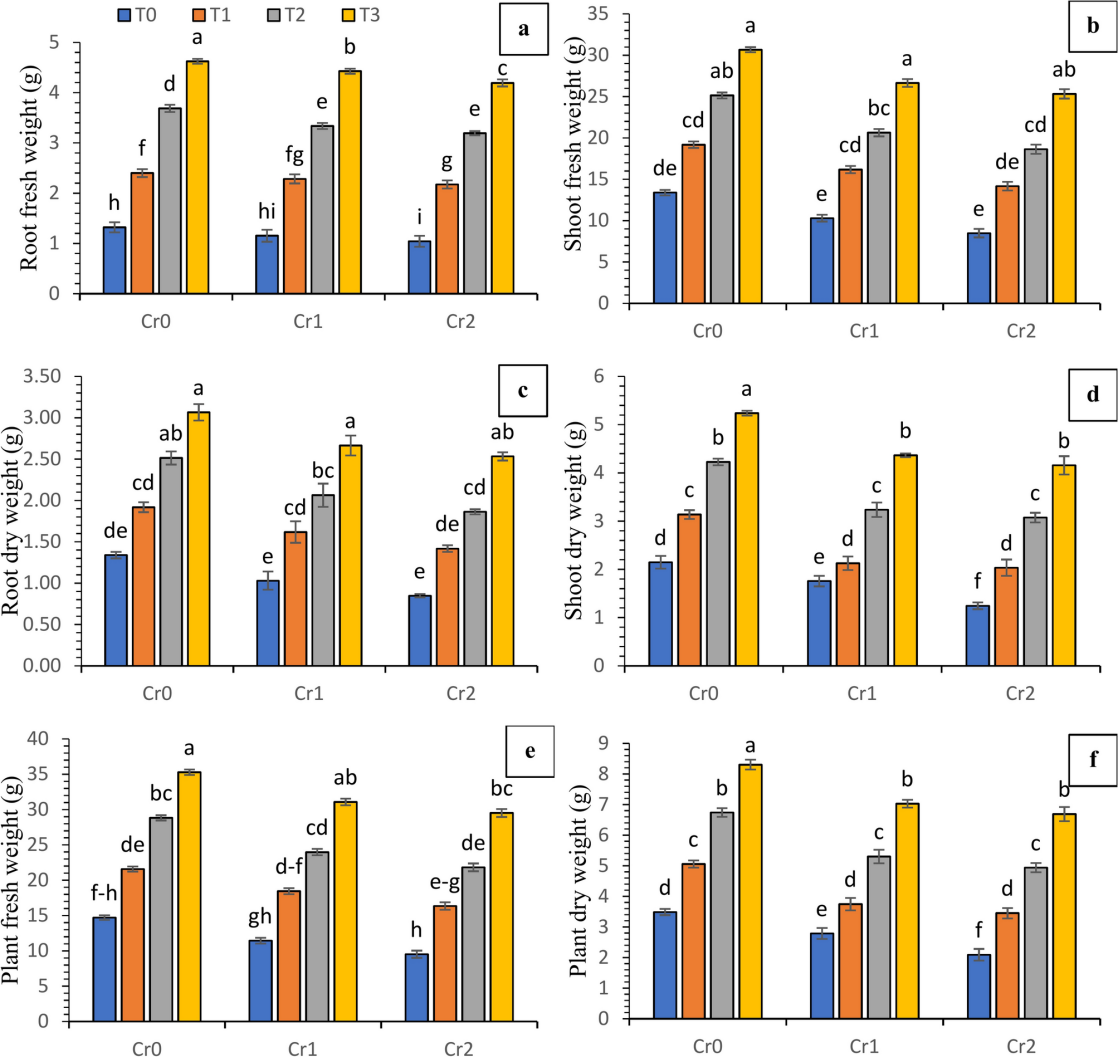
Synergistic effect of compost and Se on the biomass (fresh and dry) of chili plants grown under various concentrations of induced chromium (Cr_0_ = 0 mg kg^−1^, Cr_1_ = 100 mg kg^−1^ and Cr_2_ = 200 mg kg^−1^). Different lowercase letters represent the statistically significant difference across treatment means at *p* < 0.05. For each parameter, means with the same letter are not statistically different across treatments. T_0_ = Control; T_1_ = Se (3 µM); T_2_ = Compost (250 mg kg^−1^ of soil); T_3_ = T_1_ + T_2_; (**a**) root fresh weight; (**b**) shoot fresh weight; (**c**) root dry weight; (**d**) shoot dry weight; (**e**) plant fresh weight; (**f**) plant dry weight.

The data analysis of measured variables related to biomass (fresh and dry) showed a substantial synergistic effect of compost and Se on the chili plants (Fig. 2). The application of compost and Se resulted in a gradual increase in the biomass (fresh and dry) of shoots, roots, and plants for the T_3_ treatments, both in the presence of Cr contamination and under controlled conditions. On the other hand, treatment T_2_ resulted in a moderate increase in biomass both fresh and dry compared to treatment T_1_. The Cr-affected plants showed reduced shoot fresh weight (16.54 and 24.63%), root fresh weight (6.90 and 11.86%), plant fresh weight (15.39 and 23.10%), shoot dry weight (16.54 and 24.63%), root dry weight (22.13 and 28.75%), and plant dry weight (20.04 and 27.21%) at 100 and 200 mg kg^−1^ of Cr, respectively in comparsion with control (no chromium).

### Physiological, photosynthetic and biochemical attributes

**Fig. 3:**
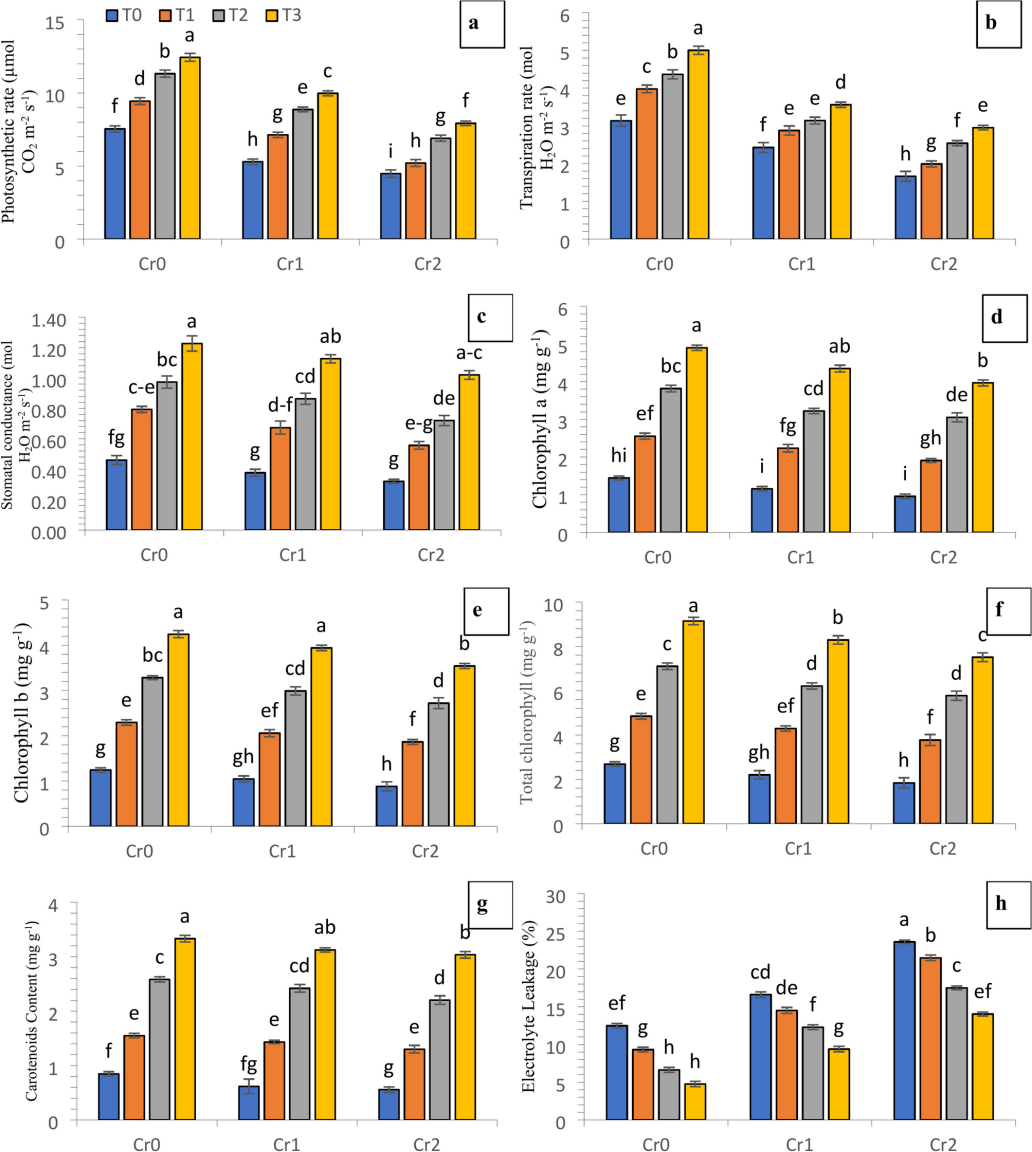
Synergistic effect of compost and Se on the physio-biochemical attributes of chili grown under various concentrations of induced chromium (Cr_0_ = 0 mg kg^−1^, Cr_1_ = 100 mg kg^−1^ and Cr_2_ = 200 mg kg^−1^). Different lowercase letters represent the statistically significant difference across treatment means at *p* < 0.05. For each parameter, means with the same letter are not statistically different across treatments. T_0_ = Control; T_1_ = Se (3 µM); T_2_ = Compost (250 mg kg^−1^ of soil); T_3_ = T_1_ + T_2_; (**a**) photosynthetic rate; (**b**) transpiration rate; (**c**) stmatal conductance; (**d**) chlorophyll a; (**e**) chlorophyll b; (**f**) total chlorophyll; (**g**) carotenoids contents; (**h**) electrolyte leakage.

The varying levels of induced Cr stress decreased the contents of chlorophyll *a* (13.82 and 22.26%), chlorophyll *b* (9.34 and 18.62%), total chlorophyll contents (11.73 and 20.57%), carotenoids levels (8.62 and 14.64%), photosynthetic rate (23.24 and 39.85%), transpiration rate (27.19 and 44.52%), and stomatal conductance (11.97 and 24.23%) and increased the electrolyte leakage (59.14 and 130.92%) at 100 and 200 mg kg^−1^ of Cr levels, respectively in comparison with the control (no chromium). Maximum change in measured traits were noted in the case of T_3_ where both compost and Se were applied for growing of chili plants in control and Cr stressed conditions (Fig. 3).

#### Water related, lipid per oxidation and enzymatic antioxidant attributes

**Fig. 4:**
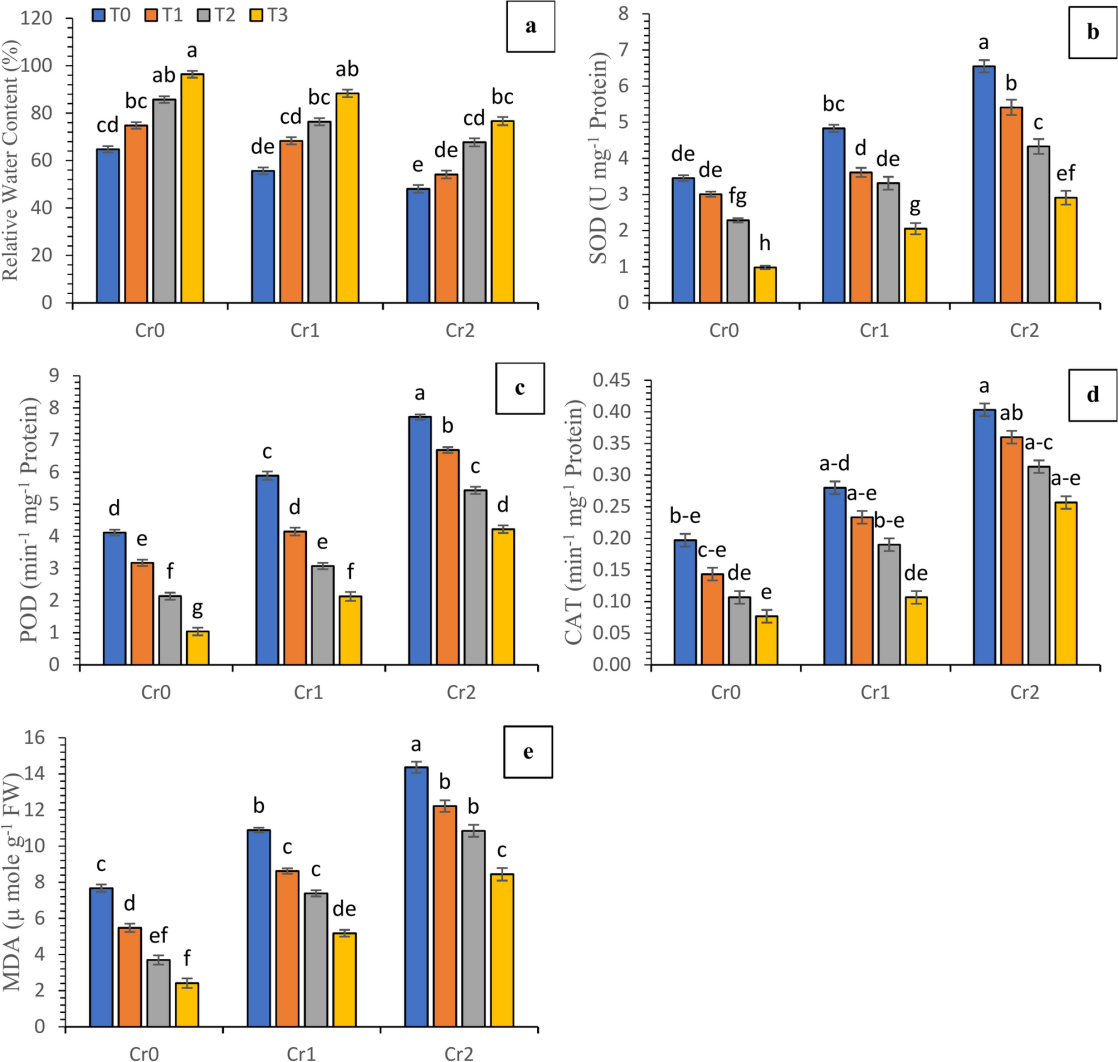
Synergistic effect of compost and Se on the enzymatic antioxidants, lipid peroxidation and water-related attributes of chili plants grown under various concentrations of chromium (Cr_0_ = 0 mg kg^−1^, Cr_1_ = 100 mg kg^−1^ and Cr_2_ = 200 mg kg^−1^). Different lowercase letters represent the statistically significant difference across treatment means at *p* < 0.05. For each parameter, means with the same letter are not statistically different across treatments. T_0_ = Control; T_1_ = Se (3 µM); T_2_ = Compost (250 mg kg^−1^ of soil); T_3_ = T_1_ + T_2_; (**a**) relative water contents; (**b**) SOD activity; (**c**) POD activity; (**d**) CAT activity; (**e**) MDA contents.

Enzymatic antioxidants, relative water contents and lipid peroxidation attributes in chili plants were altered by applying compost and Se under various levels of induced Cr (Fig. 4). Increased Cr concentration from 100 to 200 mg kg^−1^ caused the decrease in RWC (10.25–23.37%) and increased SOD (41.98–97.46%), POD (45.62–139.26%), and CAT (54.68–154.61%) and MDA (66.50–136.16%) significantly in comparsion with control (no chromium). The decreasing pattern for Cr levels were shown 200 mg kg^−1^ > 100 mg kg^−1^ > control conditions for the activities of antioxidant enzymes and lipid peroxidation,. In contrast, compost and Se treatments were noted to demonstrate a decreasing trend of T_0_ > T_1_ > T_2_ > T_3_.

### Chromium contents in plant tissues

**Fig. 5:**
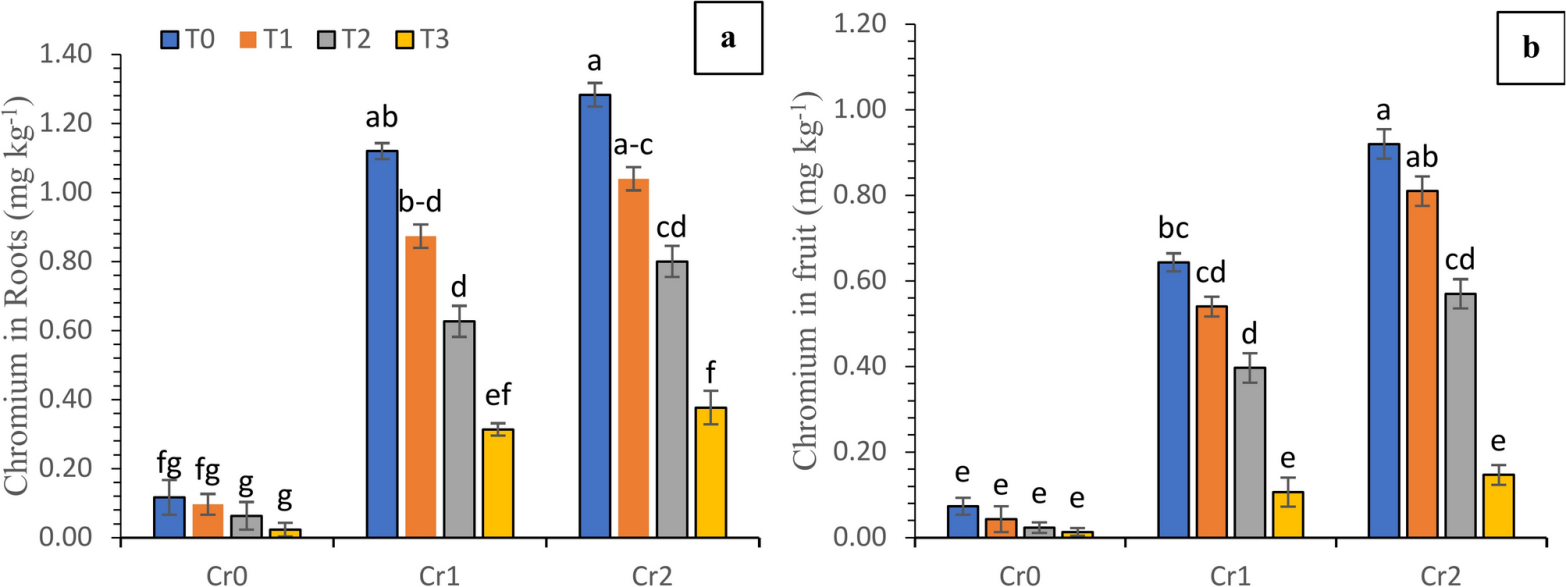
Synergistic effect of compost and Se on the chromium accumulation in plant parts of chili grown under various levels of chromium (Cr_0_ = 0 mg kg^−1^, Cr_1_ = 100 mg kg^−1^ and Cr_2_ = 200 mg kg^−1^). Different lowercase letters represent the statistically significant difference across treatment means at *p* < 0.05. For each parameter, means with the same letter are not statistically different across treatments. T_0_ = Control; T_1_ = Se (3 µM); T_2_ = Compost (250 mg kg^−1^ of soil); T_3_ = T_1_ + T_2_; (**a**) Cr contents in roots; (**b**) Cr contents in fruits.

The accumulation of chromium in the roots and fruits of chili plants was significantly influenced by the chromium levels and the different treatments of compost and selenium. This effect was observed both in plants grown under varied levels of chromium and in plants cultivated under control conditions. Under control conditions, the presence of different levels of chromium resulted in an increase in the chromium content in both the roots and fruits, compared to the control. The roots had maximum Cr content of 0.12 and 1.21 mg kg^−1^, whereas the fruit had contents of 0.07 and 0.91 mg kg^−1^ under control conditions. The use of foliar spray of Se and soil-applied compost resulted in a substantial decrease in the uptake of Cr in the roots and fruits (Fig. 5).

### Pearson correlation matrix

**Fig. 6:**
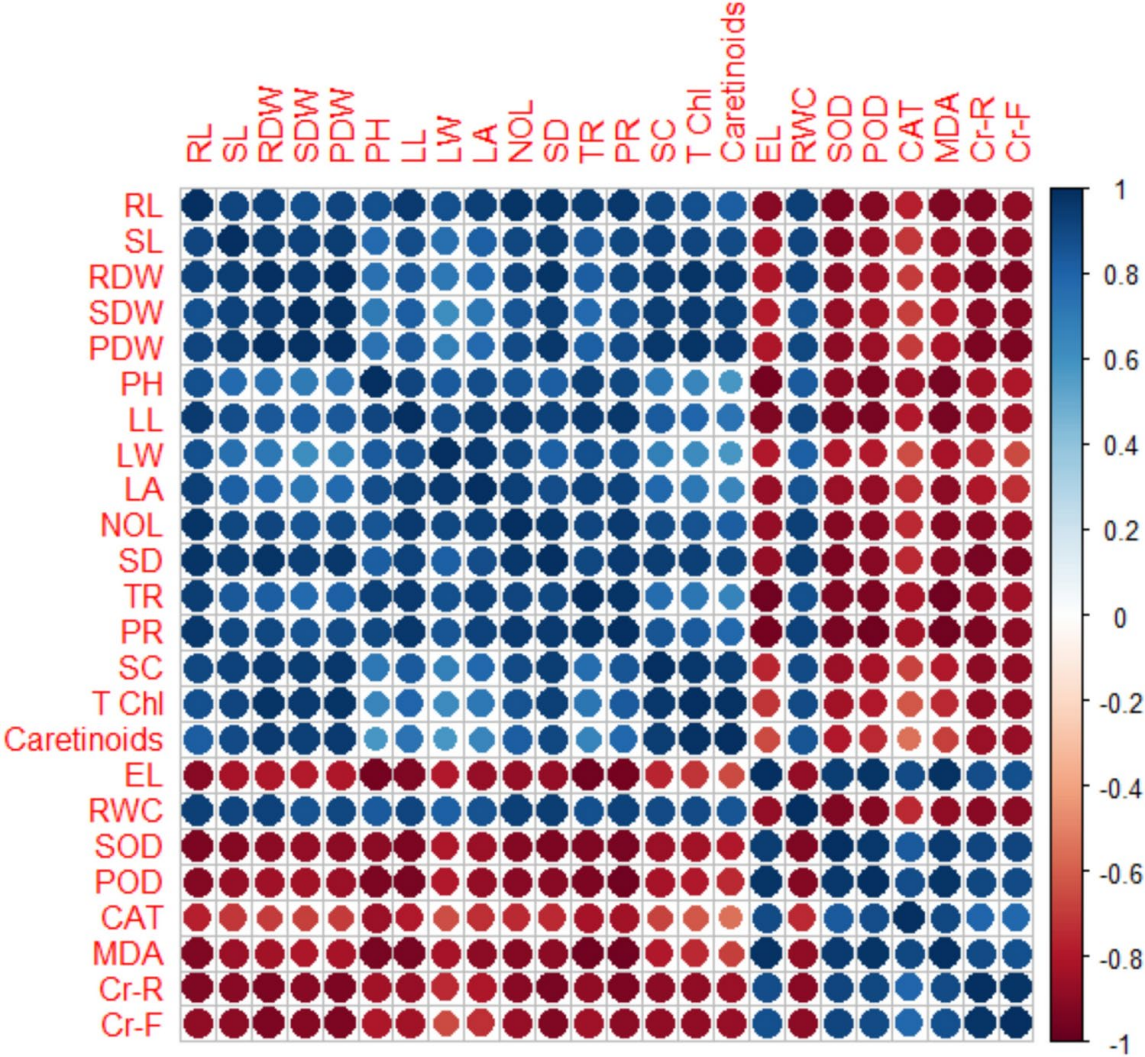
Correlation matrix of various attributes of chili by the synergistic effect of soil-applied compost and foliage-applied selenium on the chromium accumulation in plant parts of chili grown under various levels of induced chromium stress (0, 100, and 200 mg kg^−1^). *RL* root length, *SL* shoot length, *RDW* root dry weight, *SDW* shoot dry weight, *PDW* plant dry weight, *TCHL* total chlorophyll contents, *LL* leaf length, *LW* leaf width, *LA* leaf area, *SD* stem diameter, *NOL* number of leaves, *RWC* relative water contents, *SC* stomatal conductance, *PH* plant height, *TR* transpiration rate, *PR* photosynthetic rate, *MDA* malonaldehyde contents, *EL* electrolyte leakage, *SOD* superoxide dismutase activity, *POD* peroxidase activity, *CAT* catalase activity, *Cr-F* chromium in fruits, *Cr-R* chromium in roots.

Interconnections among all the measured attributes in chili plants were evident, reinforcing the validity of the conclusions regarding correlations (Fig. 6). All the growth and biomass attributes are positively correlated with the photosynthetic attributes of the chilli plants. However, the electrolyte leakage showed negative correlation with the growth and biomass attributes of the chilli plants. Enzymatic antioxidants (SOD, CAT, and POD) exhibited negative correlations with chlorophyll content, growth, biomass, and relative water content. Conversely, a notable positive correlation was observed for MDA levels, electrolyte leakage, and chromium accumulation in plant tissues. Additionally, RWC and the dry weights of chili plant roots and shoots showed favorable associations with chlorophyll content among other measured parameters.

### Principal component analysis

**Fig. 7:**
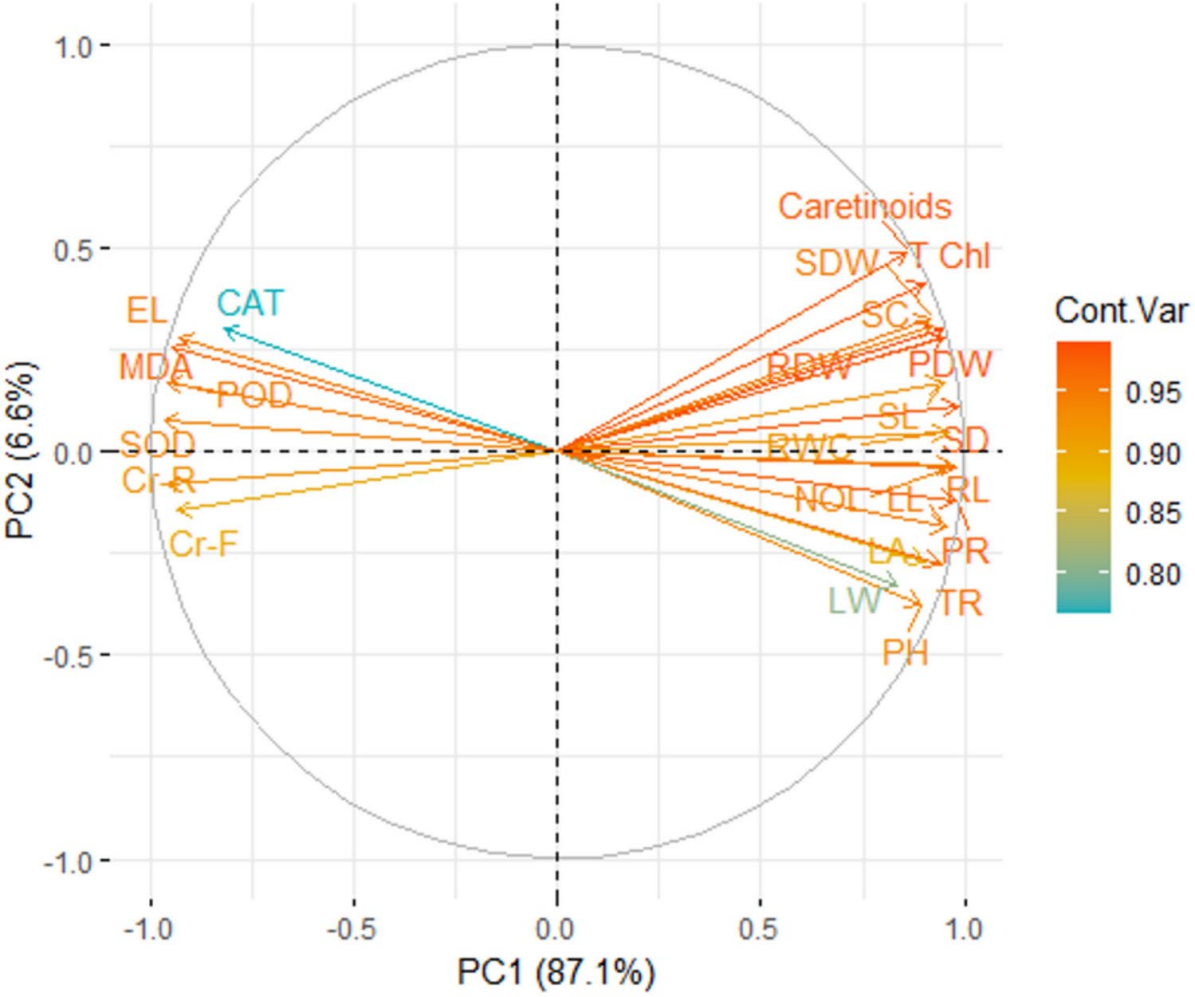
Principal component analysis (PCA) of various attributes of chili by synergistic effect of soil applied compost and foliage applied selenium on the chromium accumulation in plant parts of chili grown under various levels of induced chromium stress (0, 100 and 200 mg kg^−1^). *RL* root length, *SL* shoot length, *RDW* root dry weight, *SDW* shoot dry weight, *PDW* plant dry weight, *TCHL* total chlorophyll contents, *LL* leaf length, *LW* leaf width, *LA* leaf area, *SD* stem diameter, *NOL* number of leaves, *RWC* Relative water contents, *SC* stomatal conductance, *PH* plant height, *TR* transpiration rate, *PR* photosynthetic rate, *MDA* malonaldehyde contents, *EL* electrolyte leakage, *SOD* superoxide dismutase activity, *POD* peroxidase activity, *CAT* catalase activity, *Cr-F* chromium in fruits, *Cr-R* chromium in roots.

The chili plants are grown on Cr contaminated soil along with the Se application and compost treatments depicted an association between Cr levels, and various observed attributes by principal component analysis (Fig. 7). The primary component (PC1) accounts for the largest proportion of variability, encompassing over 87.00% of the total variance, while PC2 represents 6.60% of the variability among the assessed variables. PC1 exhibits a positive correlation with chromium, MDA, SOD, POD, and CAT, indicating a positive interrelation among these factors. Variables that are close together or point in a similar direction are positively correlated. The color gradient (from yellow to red) indicates the contribution variance of each variable to the principal components, with warmer colors showing higher contributions. Conversely, factors associated with SOD, CAT, and POD show a slight negative correlation with growth and biomass attributes. Notably, parameters related to growth and biomass exhibit a robust positive relationship with PC1 variables.

### Heat map analysis

**Fig. 8:**
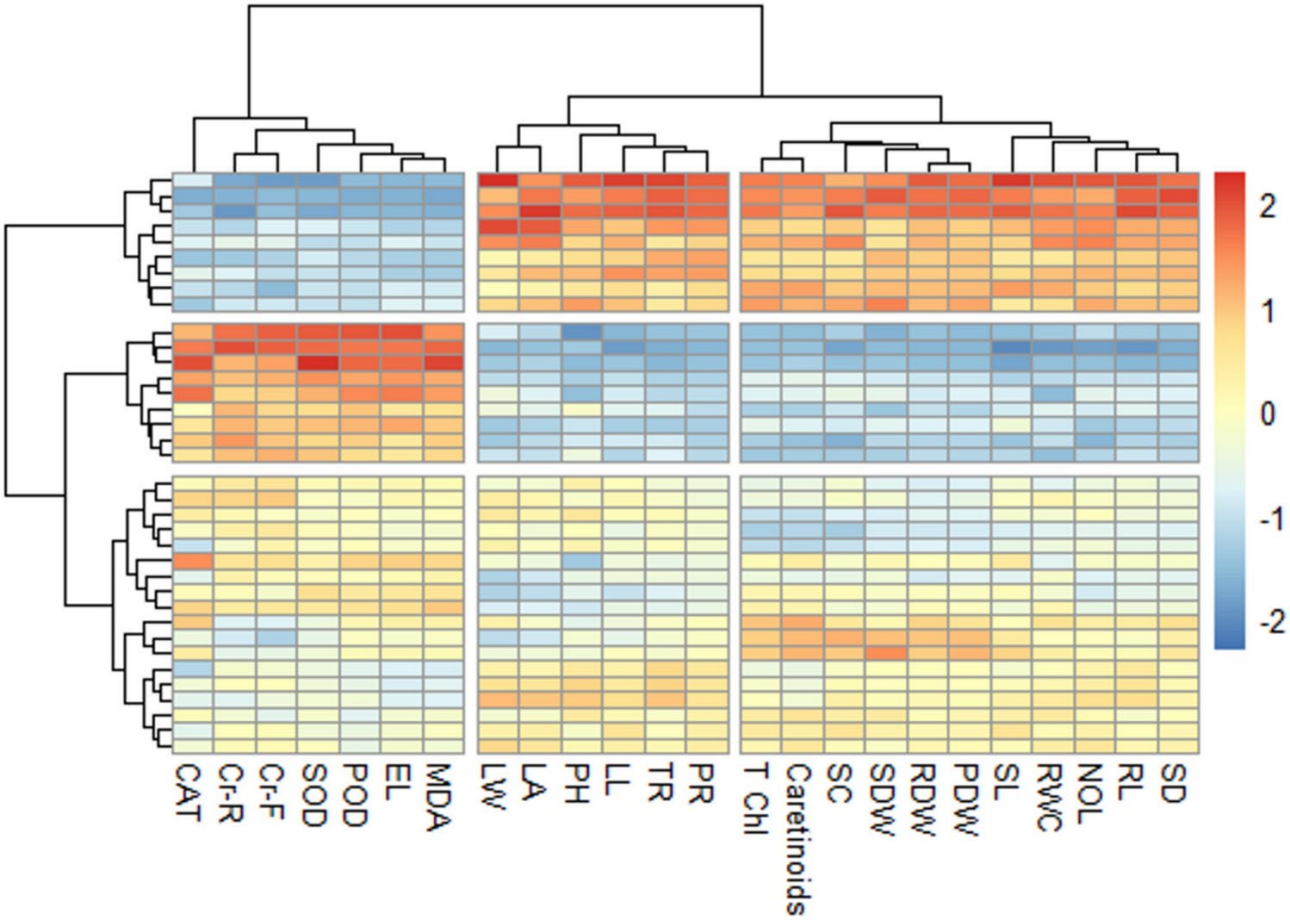
Heat map plot of various attributes of chili grown by synergistic effect of soil applied ompost and foliage applied selenium on the chromium accumulation in plant parts of chili grown under various levels of induced chromium stress (0, 100 and 200 mg kg^−1^). *RL* root length, *SL* shoot length, *RDW* root dry weight, *SDW* shoot dry weight, *PDW* plant dry weight, *CHL* total chlorophyll contents, *LL* leaf length, *LW* leaf width, *LA* leaf area, *SD* stem diameter, *NOL* number of leaves, *RWC* relative water contents, *SC* stomatal onductance, *PH* plant height, *TR* transpiration rate, *PR* photosynthetic rate, *MDA* alonaldehyde contents, *EL* electrolyte leakage, *SOD* superoxide dismutase activity, *POD* peroxidase activity, *CAT* catalase activity, *Cr-F* chromium in fruits, *Cr-R* chromium in roots.

The interaction among various traits of chili plants grown in chromium-spiked soil was assessed using a heatmap histogram (Fig. 8). The intensity of each color reflects the magnitude of the response, with mid-range colors like yellow depicting neutral or zero values. Hierarchical clustering on both the rows and columns organizes variables and treatments into groups based on similarity in response patterns, as shown by the dendrograms along the top and left side. A negative correlation was observed between MDA concentration and relative water content, while a positive correlation was found between MDA concentration and chromium levels in the roots and fruits of chili plants. Electrolyte leakage exhibited a significant correlation with chromium levels in plant roots. Additionally, a significant positive association was observed between growth traits when selenium and compost were applied together. Furthermore, chromium uptake showed a negative correlation with antioxidant enzymes (SOD, CAT, and POD).

## Discussion

The existence of industrial waste and effluents has resulted in significant ecological risks on arable land, primarily due to elevated concentrations of heavy metals^32^. These metals have the ability to cause damage to plants, which have been provided with numerous essential antioxidant mechanisms. The results of the present study indicated that the growth and biomass attributes of chili plants were substantially affected by the modulation of antioxidative and lipid per oxidation potential as a result of varying levels of Cr stress. The growth traits of chile were found to be reduced as the result of the elevated concentrations of metals and metalloid stress in previous research^33^. The reduction in cell elongation observed under limited water conditions can be attributed to the stress induced by the metals^6^. Specifically, higher levels of Cr have been found to impede water movement from the xylem to the neighboring cells^34^. Abiotic stress conditions, including metal stress, have been found to induce a decline in turgor pressure and a decrease in the rate of photosynthesis. This metallic stress impedes the expansion of leaves^32^. There was evidence that Cr contamination in mungbean plants slowed down the growth of both roots and shoots, decreased biomass, and lowered the number of leaves, branches, and twigs^35^. Applying biochar or compost as soil amendments at an optimal concentration enhances the morphological and developmental traits of maize plants^36,37^ by mitigating the adverse effects of metal-induced stress in tannery industry-polluted soils^38,39^. The findings revealed that the synergistic effect of selenium and compost on plants subjected to higher levels of chromium resulted in notable enhancements in leaf size, growth and biomass traits (Figs. 1 and 2). Similarly, the contribution of selenium and carbon-containing compounds (compost) in the cell division leads to an augmentation in plant height and intermodal elongation^40–42^. Conversely, applying compost to the soil has been found to restrict chromium uptake in plant cells. The observed enhancement can be ascribed to the substantial role of compost, which has effectively adsorbed the bulk of chromium and consequently decreased its availability and potential risks for plant growth^19,43–45^. The findings of Juleel et al.^44^ were consistent with regard to the enhancement of plant growth and biomass following the application of compost or vermicompost on under abiotic stress. The research conducted by Saleem et al.^46^ revealed that the direct application of compost and biochar is a regulatory mechanism for cell division and cell elongation enhancement in plant under the abiotic stress.

The photosynthetic characteristics of many field and vegetable crops exhibited a linear reduction when exposed to induced chromium contamination. The reduction in the photosynthetic attributes in chili plants was attributed to the presence of higher levels of Cr (Fig. 3). The findings of Sharma et al.^47^ depicted an observed decrease in porphobilinogen deaminase activity and subsequent reduction in chlorophyll levels as a result of stress induced by Cr. The present study revealed that chili plants exposed to elevated chromium levels showed increased electrolyte leakage, a direct sign of stress vulnerability, compared to control plants. (Fig. 3). The excessive production and buildup of reactive oxygen species (ROS) have been identified as potential causes for the increased lipid peroxidation, protein insufficiency, and considerable growth reduction associated with DNA damage^48,49^. The concurrent utilization of compost and selenium may yield synergistic outcomes in terms of better plant growth in Cr-contaminated plants as shown in Fig. 1. According to Malik et al.^19^, compost can improve nutrient accessibility, promoting healthier plant tissue development. According to Lanza and Dos Reis^50^, it was found that selenium can facilitate cellular activities and mitigate oxidative stress. These characteristics may contribute to enhanced cellular membrane integrity and reduced electrolyte leakage. The findings of the present investigation indicate that the application of induced chromium significantly influenced the photosynthetic capacity of chili plants than the control group plants. The research revealed that exposure to chromium-induced stress reduces chlorophyll synthesis, resulting in a subsequent decline in the photosynthetic rate (Fig. 3). In the present study, under both chromium contamination and control situations, applying compost and selenium resulted in elevated levels of chlorophyll and carotenoids. The increase in chlorophyll content and photosynthetic parameters can be ascribed to the synergistic effects of enhanced nutritional supply, antioxidant defense, stomatal management, and enzymatic activity, culminating in the observed enhancement in chili plants. The study conducted by Sami et al.^51^ revealed similar outcomes were observed when spinach plants exposed to chromium stress were treated with combined application of Se and biochar. This application potentially reduced oxidative damage to chlorophyll pigments by increasing the concentration of carotenoids. According to Jahan et al.^52^, this process contributes to enhancing carotenoid and chlorophyll levels by limiting the electrolyte leakage. Under conditions of induced chromium, the use of selenium and compost may have resulted in enhanced chili physiological variables, including increased total chlorophyll content. The application of selenium through foliar application resulted in a decrease in the negative effects on the process of photosynthesis under various levels of Cr. The beneficial impacts of selenium on the process of photosynthesis, in both normal conditions and in the presence of chromium, can be attributed to its ability to mitigate oxidative stress, augment enzyme activities (glutathione peroxidase) which aids in the neutralization of reactive oxygen species generated during photosynthesis), and facilitate metabolic processes^53^. Despite being non-essential, the interactions of Se with many physiological processes have been found to enhance photosynthesis and bolster plant resistance^51^. Metal-stressed plants exhibit enhanced photosynthetic efficiency due to the elevation of several physiological, biochemical, and metabolic processes^44,54^. This improvement is observed through an increase in total chlorophyll levels and the activation of antioxidative mechanisms, which are facilitated by applying selenium and compost (Fig. 3). In the context of soil culture trails, the presence of chromium has been observed to significantly influence the activity of antioxidant enzymes as similar findings were observed in the current study (Fig. 4). In soils contaminated with pollutants, the enzymatic actions of several parameters such as catalase (CAT), peroxidase (POD), superoxide dismutase (SOD), and malondialdehyde (MDA) were noted indicating a linear increase. The enzymatic activities of catalase, superoxide dismutase, and peroxidase have been observed to facilitate the conversion of hydrogen peroxide (H_2_O_2_) into water (H_2_O) and molecular oxygen (O_2_) inside a cell. The observed enhancement in the antioxidant defense system, leading to increased activities of SOD, CAT, and POD, and improved plant growth under chromium stress, can potentially be attributed to the significant role of selenium and the absorption capacity of compost^55^ The adverse effects of selenium element, caused by the excessive generation of reactive oxygen species (ROS), have been found to play a crucial role in the activation of enzymes involved in the detoxification of ROS, leading to membrane leakage, and the prevention of toxic hydroxyl radicals^51^.

Significant differences were seen in the chromium accumulation in both the fruit and roots of chili plants across the various treatments as shown in Fig. 5. The root had the highest concentration of chromium compared to the fruit, suggesting a potential correlation with metallic elements. According to the studies conducted by Zewail et al.^56^ and Abdullah et al.^57^, the resemblance in structure between chromium and some key elements can provide challenges in mineral nutrient digestion and translocation in plants. Plant nutrient uptake decline can also be attributed to reduced root development and surface area^58^, as a similar observation was also noted by the correlation analysis mentioned in Fig. 6. Chromium uptake in plants is commonly observed through a diverse range of methods^59^. Our investigation has shown that there is a direct correlation between higher levels of chromium in plants and increasing concentration of chromium in the soil. In addition, it is generally defined that plants primarily obtain Cr (III) through passive diffusion. At the same time, active processes in plants typically facilitate the uptake of Cr (VI), which involves carriers responsible for transporting essential anions such as sulphate (SO_4_^−^)^58^. The utilization of compost and selenium has been found to significantly reduce the levels of chromium in its accessible form^51^. According to Iqbal et al.^60^, it was observed that the increased efficacy of compost may be attributable to chromium adsorption onto the surface of the compost. The utilization of compost and selenium in conjunction has been found to enhance plant growth by reducing chromium absorption in leaves, thereby decreasing the bioavailability of Cr in chili plants. Similar finding were observed in the lettuce plants^61^. This comprehensive approach promotes sustainable crop productivity and mitigates the potential of Cr stress in the chili. In summary, the concurrent application of selenium and compost demonstrated higer efficacy compared to individual treatments in mitigating Cr uptake in the aboveground and belowground portions of plants. These outcomes suggest enhanced resistance or tolerance to Cr-induced stress.In future research, it is recommended to extend the investigation of the combined effects of different factors to include environmental variables as well. This expansion will contribute to a more comprehensive understanding of the interactions between various factors and their influence on sustainable agricultural systems.

## Conclusion

The present study’s findings demonstrate that exogenously-applied Se and soil applied compost, which effectively attenuated the Cr stress by improving the growth, biomass and antioxidative potential of chili plants. The enhanced photosynthetic and physiological attributes were ascribed to the synergistic effect of compost and Se in improving the antioxidative potential and limiting the lipid per oxidation (MDA contents) of chili plants. The combined approach of Se and compost played a crucial role in limiting the uptake of Cr ions in the roots and fruits portion of chili plants. Based on the experimental work, it can be concluded that the combined application of compost and Se is sustainable approach for mitigation of Cr stress in chili cultivation. Furthermore, applying compost in combination with other amendments and various salts of Se can open new avenues of environmentally friendly approaches to growing and enhancing chili cultivation produced in metal-stressed soil on a long-term basis. The effects of compost and Se on chili productivity has implications for researchers, conservationists and commercial farmers.The future studies must focus on the concentration of Se in the edible parts and also its potential helath risk index to observe the Se biofortication. However, before recommending it commercially, the economic factors must be analyzed for better viability.

## Data Availability

The datasets analyzed during this study are included in this manuscript.

## Declarations

### Statement on guidelines

All experimental studies and experimental materials involved in this research are in full compliance with relevant institutional, national, and international.

guidelines and legislation.
